# Exploring and addressing the sexual and reproductive health and other related rights of transgender women in Bangladesh: A mixed methods protocol under the policy analysis framework

**DOI:** 10.1371/journal.pone.0306051

**Published:** 2024-07-26

**Authors:** Samira Dishti Irfan, Masud Reza, Mohammad Niaz Morshed Khan, Sharful Islam Khan

**Affiliations:** Programme for HIV and AIDS, Infectious Diseases Division, International Centre for Diarrhoeal Diseases Research, Dhaka, Bangladesh; Children’s Mercy Hospital Kansas, UNITED STATES

## Abstract

**Introduction:**

Transgender women (*hijra*) in Bangladesh are declared as a separate gender category by the Government. However, research revealed that they experience transphobia, which could potentially affect their physical and mental health outcomes, and their access to SRHR-related care. This warrants an exploration of their SRHR-related rights issues, particularly using a community-engaged approach. Moreover, it is crucial to operationalize these findings into actionable policies and practice. This study aims to explore and address the SRHR and other rights-related challenges experienced by *hijra* under the framework of policy analysis.

**Methods:**

The study population will include *hijra* in four selected service centers in Dhaka, Bangladesh. In the first phase, evidence will be generated through desk review and mixed methods research. The desk review will consist of reading and analyzing literature to understand the difference between policy and reality. For the quantitative component, a first-come-first-serve sampling approach will be used on a total sample size of 296. This will be complemented by the qualitative component, which will entail in-depth interviews, focus groups and key informant interviews. Moreover, life case histories will be conducted for particularly compelling cases. These findings will be collectively analyzed through the policy analysis framework, to analyze the differences between the policy and reality, which will ultimately generate a lay summary for stakeholders. Univariate and multivariate analysis will be used for the quantitative component whereas thematic analysis will be used for the qualitative component. In the second phase, the findings from the lay summary will be shared with stakeholders and *hijra* community members through a series of discussions.

**Discussion:**

There are a few limitations of the study. In particular, this study consists of various activities which may require substantial time and effort to complete. Additionally, this study merely goes up to the policy recommendation formulation stage, as opposed to formulating an intervention design. Moreover, the findings will be disseminated through various platforms, including dissemination seminars, scientific articles and the study report.

## Introduction

### Transgender populations and their positionality in relation to their rights

Transgender populations constitute an umbrella concept encompassing a broad spectrum of gender-diverse populations [1]. It is worth noting that, even though they are labelled as transgender populations, each population group has its own set of nuanced cultural differences. For instance, two-spirited populations in Native American regions identify as having both a masculine and feminine spirit [2]. Whereas, kathoeys (sometimes called ladyboys) in Thailand are born as male but identify as a woman, and undergo bodily modifications to reflect a feminine gender expression [3]. Similarly, in the Indian subcontinent, hijra (male-to-female transgender populations who belong to a specific hijra sub-culture) are categorized as a separate gender category within the country’s legal frameworks [4]. Although this list is not exhaustive, all transgender populations are defined as populations whose preferred gender identity does not align with their sex assigned at birth.

Each individual is entitled to the fundamental right of pursuing a life of human dignity irrespective of their socio-demographic status, including their sexual and gender identity, expression and orientation. In line with this principle, several governments around the world have imparted that degree of respect towards these populations, sometimes to the extent of acknowledging them as a separate gender category. This respect has also been transposed to the societal spheres where transgender populations are normalized such as many Western settings, Thailand, Vietnam, etc. However, not all transgender populations have the freedom to exercise their rights, as many countries continue to face ongoing attacks on the medical, legal and socio-cultural rights of these populations [5].

However, in several countries, transgender women are entrapped in a limbo where their rights are legally recognized, although their socio-structural environments are not conducive to them exercising their rights [6, 7]. Therefore, their gender identities are not acknowledged within the socio-structural framework of their countries, thus exacerbating their risk of gender-based violence, discrimination and harassment. According to the recent UNAIDS report, transgender women experience considerable stigma, discrimination and violence at the structural level (i.e. in laws, policies and institutionalized practices) and societal level (i.e., mistreatment, rejection, lack of gender recognition, etc.) [8].

This is corroborated by evidence in settings even as progressive as the San Francisco and Los Angeles areas, where 45.8% of the transgender women in San Francisco experienced abuse or discrimination at least once in their lifetime. Furthermore, a systematic review revealed that the pooled prevalence of discrimination and transphobia ranged from 2–41.8% [9]. This phenomenon was also found to be pervasive in several settings in the Asia Pacific region. A study among transgender women in South Korea revealed that transgender women who avoided healthcare were 1.9 times more likely to attribute it to discrimination based on their gender identity, than those who did not avoid healthcare [10]. Similarly, qualitative evidence from China indicated that transgender women faced high rates of social rejection and discrimination, which was manifested in many ways including self-identification issues, compromised healthcare access and culturally shaped expectations of reproduction [11]. Moreover, within the immediate region, a qualitative study conducted in Chennai, India revealed that transgender populations experience stigma and discrimination in various settings such as education, healthcare, changing national ID cards, getting bank loans, etc. [12].

### The intersection between the gender recognition of transgender women in Bangladesh and their sexual and reproductive health and rights (SRHR)

The paradigm shift of accommodating alternative gender identities has been identified in Bangladesh, where the Government of Bangladesh (GoB) has legally acknowledged transgender women (locally known as *hijra*) as a separate gender category since 2013, for which the gazette notification was issued in January 2014 [13]. This has provided them the platform to exercise basic citizenship rights such as voting using their correct identity (i.e., as a hijra) and indicating their gender on national identification documents.

However, research evidence in Bangladesh indicated that their rights are often yet to be realized institutionally and societally, as they remain subjected to health and other rights infringements, namely healthcare access challenges. As the local literature indicates, Bangladesh follows traditional views of gender that adhere to heteronormativity, influenced by a combination of government rules and societal expectations [14]. Most of the studies on hijra based in Bangladesh have shown that hijra emerge from lower socioeconomic backgrounds, as they are unable to attain mainstream employment opportunities, which constitutes another infringement in their rights [15]. As local evidence indicated, *hijra* were reluctant to visit healthcare facilities due to experiences and anticipatory fears of discrimination, denial of services and neglect [16]. These circumstances could potentially exacerbate their vulnerability to sexually transmitted infections (STIs), gender dysphoria, and other sexual and reproductive health and rights (SRHR) conditions; as well as other physical and mental health outcomes [8, 17–19].

### Existing research on SRHR and intervention gaps: What we know vs. what we need

There is a growing body of evidence about the *hijra* population, particularly in the domain of healthcare access barriers. However, the existing research and interventions have remained limited to understanding and addressing HIV and STIs, without accounting for their other health and rights issues, including their SRHR. In particular, gender rights and gender-inclusivity are overlooked in the existing research framework, especially in Bangladesh and other similar settings. To ensure optimal rights-related outcomes for *hijra*, it is essential to expand this knowledge base with a holistic and comprehensive understanding about their lived experiences, their rights status and violations, and existing barriers to achieving SRHR, and to some extent, other citizenship rights. Without further research on the rights-related aspect of SRHR, it would be difficult to develop a holistic intervention that actively works on giving *hijra* the platform to freely exercise their rights, regardless of their gender identities and expressions. Thus, this context necessitates research that can provide insights on prominent rights-related sufferings experienced by *hijra*, so that these rights infringements receive deeper attention in future and extant intervention activities either by government entities, the private sector or by donors.

### Taking the evidence from the paper to the ground: Justifications for this research

Research has limited value unless operationalized into actions that can be incorporated into policy and routine practice [20–22]. Rather, experts highlight the importance of embedding participatory activities within the research itself to ensure that findings come to fruition [23]. In the domain of transgender research, there is currently an ample body of literature about the lived struggles and socio-structural challenges experienced by hijra. However, there remains a paucity of efforts to synthesize evidence and devise policy recommendations in consultation with stakeholders. Yet, in the context where transgender women are experiencing rights infringements, it is integral to uphold their rights through policy and practice. Therefore, this research with a policy translation component is warranted, where stakeholder discussions constitute an integral facet of the research. Given the paucity of information about the existing rights status, violations and lived experiences in relation to their SRHR and other related citizenship rights, these knowledge gaps could be supplemented through research and shared with relevant stakeholders.

This study aims to respond to the research question, “What are the challenges faced by *hijra* in terms of their SRHR-related rights and how can they be addressed to formulate rights-responsive SRHR interventions for *hijra*?” This article aims to describe the study protocol that addresses these research questions which will be addressed through the following objectives:

1. To understand and measure the current status of human rights conditions and lived experiences in relation to the *hijra*’s ability to protect and practice their gender, sexual and reproductive rights, and related human rights agenda;
2. To explore the gaps in policy and grounded reality of human rights conditions and lived experiences of *hijra;*
3. To identify priority areas related to their rights that necessitate interventions to uphold human rights including SRHR-related rights;
4. To formulate evidence-based policy recommendations which can lay the foundation for rights-based SRHR interventions for *hijra;*

### Outcome variables

This research contains five main outcome variables adapted from the Guttmacher recommendations of SRHR indicators for the Sustainable Development Goals. However, not all of the recommendations were utilized for this study as many were not applicable to the local context. Moreover, since policy analysis is a major theoretical facet underpinning this study, a few outcome variables have been added corresponding to the principles of the policy analysis framework, such as the ability to freely express their gender identity and preferences in relation to the gender policy (outcome variable d and e), and negotiate their rights as inscribed in the gender policy (outcome variable c).

- Episodes of discrimination, stigma and other gender-based rights infringements in public or private healthcare facilities;
- Episodes of discrimination, stigma and other gender-based rights infringements in other mainstream settings (banks, employment sector, education, etc.);
- Ability to negotiate their own rights in a mainstream forum outside their own *hijra* community;
- Ability to safely express their gender identity and choice of sexual partner;
- Basic knowledge about SRHR;
- Episodes of rights infringements which have contradicted the separate gender declaration

## Materials and methods

In response to the research questions, this study will follow the cross-sectional design of the descriptive category of non-experimental design. This cross-sectional design will follow the mixed methods approach, consisting of qualitative and quantitative inquiry. The following section will describe the steps taken and the theoretical propositions applied in this research.

To respond to the research question and align with the study objectives, this study will consist of two phases: evidence generation (7 months), primarily consisting of desk review and mixed methods inquiry, and formulating policy recommendations, where findings will be deliberated with stakeholders (7 months). To carve the pathway for sustainable changes in the existing policy, the policy analysis framework [24] will be applied.

### Applying the policy analysis framework: Discerning the theory and reality

To inform culture-sensitive SRHR interventions for *hijra*, a robust understanding of the existing policy and its implementation in the reality needs to be established. Scholars acclaimed this theoretical proposition as a “social pillar of sustainable development” [25] because of its ability to inform long-term policy, even to the state/national level. For discerning the policy and reality, Centre for Disease Control (CDC) and other theoretical scholars underscored the use of the policy analysis framework which will consolidate our understanding of the third gender declaration and identify the areas where their rights are not being exercised properly. The policy analysis framework embodies the following characteristics:

1. Providing a critical foundation for analyzing policies
2. Encapsulating the different dimensions of policy contexts which include the history, politics, everyday lived experiences and diverse knowledge about the subject matter
3. Generating transformative knowledge, insights, policy solutions and actions which would have not been otherwise achieved by other frameworks.

Therefore, the policy analysis framework would be applied to not only consolidate our understanding of the existing declaration of their gender category, but also to help identify the areas where their rights are not being exercised properly. According to the CDC, the key steps in the policy analysis framework include:

1. Identifying the problem or issue
2. Identifying an appropriate policy solution
  - Identifying and describing policy options
  - Assessing policy options
  - Prioritizing policy options
3. Developing a strategy for furthering the adoption of a policy solution

The algorithm for the policy analysis framework has been depicted in Table 1. The descriptions of the components of the policy analysis have been adapted from the CDC, and explained as per the objectives of our study.

**Table 1:** Detailed descriptions of the application of the policy analysis framework in the proposed study.

Step of policy analysis	Description	Mechanism of application for our study
Identifying the problem	We need to synthesize data on important characteristics of the issues such as the burden, frequency, severity, and scope in relation to the existing policy at hand.	Evidence will be generated through two pathways: (a) desk review of the available policy documents, scientific literature and media reports; and (b) empirical evidence through quantitative and qualitative inquiry about their lived experiences, rights status, etc.
Identifying the appropriate policy solution	Identify and describe policy options by reviewing literature on the topic, surveying best-practices and eliciting the perspectives of the participants Assessing policy options based on the effectiveness of these best practices Prioritizing policy options based on the assessment	We can elicit best-practice policy revisions through the following two pathways: (a) interviewing *hijra* and other stakeholders about their perceived recommendations for informing a more gender-inclusive policy; (b) consulting the global literature to learn more about best-practices for gender-inclusive policies. Based on this, we will prioritize the best-practices that are more suitable for our study context and findings.
Developing a strategy for furthering the adoption of a policy solution	A strategy needs to be defined for making sure that the policy gets enacted and implemented. This stage could consist of clarifying operational issues, identifying and educating stakeholders, and sharing relevant information.	Our strategy for operationalizing evidence into action would be to conduct a series of stakeholder discussions. We would educate them about the complexities experienced by *hijra*, and share the relevant findings.

As indicated in Table 1, the policy analysis framework will be applied throughout the study in different ways. As per the principles of the framework, the evidence will be the synthesized through the two main pathways of generating empirical evidence and conducting a desk review.

The study will primarily analyze the history of the issuance of the law and gazette notification, along with documents indicating the rights that hijra are entitled to, e.g. national documentation, banking, employment laws, protection laws, etc. These policies will be identified through an extensive document review, as well as key-informant interviews with policy level stakeholders. The policy analysis will respond to the fundamental question of whether the existing policies align with their ability to exercise their rights in the grounded realities. This would be complemented by the empirical evidence, which would reveal insider perspectives of whether their rights are actually being exercised on the ground. Identifying these discrepancies constitutes a key component of the policy analysis.

### The continuum of evidence to action

Policy action and change need to be achieved, with the intended scope and audience being the policy and programmatic stakeholders on one hand, and the *hijra* community members on the other [26]. This approach would be helpful for transposing the experiences of *hijra* community members into usable knowledge which could influence practice.

In line with this proposition, we will undertake a series of discussions with hijra community members and stakeholders. Although we plan to initially present our summarized findings from the first phase, this will segue into multiple interactive discussions. These discussions will start by free-listing the research findings where priority areas for interventions will be gradually identified after stakeholders and community members share their opinions and feedback based on their real-world experiences [23]. Through this approach, the discussions will funnel down from a broader range of research findings to narrow, focused discussions about subsequent steps for addressing the rights issues of *hijra*. Eventually, all hijra community members and stakeholders will convene in a single setting to collaboratively finalize the list of priority issues to be intervened and eventually formulate policy recommendations together. It is worth noting that hijra community members will play an instrumental role in reviewing and validating the findings, while complementing the shared findings with their own grounded perspectives. The policy recommendations generated from these discussions will be shared back with hijra community-based organizations so that they can be used to further their own hijra rights advocacy agenda.

### Staffing structure, recruitment and training of research team members

The field research team will consist of a Research Fellow, a Field Research Assistant (FRA), and a Data Management Assistant (DMA). While the Research Fellow will be responsible for data collection, analysis, field management, report writing and facilitating the stakeholder discussions, the FRA will primarily undertake the data collection and support the analysis process. Meanwhile, the DMA will assist with the data collection where necessary by ensuring data entry and data cleaning. After being onboarded onto the team, they will undergo a rigorous training workshop lasting for a total of five working days. The training will be facilitated by experts in SRHR, gender issues, mixed methods research, and the complexities faced by key populations, including *hijra*. The training workshop will cover important issues which are delineated in the Table 2:

**Table 2:** Training schedule for the staff about the study.

Day	Training issues
**Day 1**	• Transgender populations and their SRHR complexities • The history of the transgender gender declaration in Bangladesh
**Day 2**	Study objectives, scopes and methodologies
**Day 3**	• Conducting quantitative and qualitative research • Policy analysis framework
**Day 4**	• Data collection, management and analysis techniques • Ethical considerations • Facilitation of stakeholder discussions
**Day 5**	• Questions and answer sessions • Open discussions with facilitators • Role plays of interviews and obtaining consent

It is also worth noting that this basic training workshop will be compounded by ongoing refresher trainings throughout the project period, depending on the needs. It is hoped that this approach will encourage a constant learning environment, thus creating dynamism among the research team.

### Study population and sites

The study populations will primarily include *hijra* aged between 18–60 years old residing in Dhaka city. Dhaka city has been chosen as the study site because of its representativeness relative to other regions in terms of the hijra culture, intervention coverage and socio-structural struggles experienced by hijra. Although they will be primarily recruited through the HIV prevention interventions, we will be conducting a lot of the interviews at their residences. This could open avenues for interviewing participants who are left out of the intervention coverage. This would also help alleviate any bias associated with the intervention. It is also worth noting that, we will be enlisting assistance from the *hijra* to ensure that the data collected by the researchers have been captured and interpreted correctly during the member checking sessions, otherwise violations of all types of rights will end up underreported, considering the sensitivity of this topic. During the member checking sessions, the hijra will be compensated for their time through a small amount of money and refreshments.

In addition, we will also be including stakeholders as our study participants in both phases. Although the stakeholder list will be identified through stakeholder analysis, some stakeholder groups may include: representatives from ministries, policy planning stakeholders, religious leaders, human rights activists, journalists, healthcare practitioners, etc.

In addition to applying stakeholder analysis, we will also choose the stakeholders who have shown interest in acting towards the betterment of key populations (KPs) at risk of HIV (which include *hijra*) in previous research and activities.

To be eligible to participate in the study, the participants need to fulfil the following inclusion criteria:

- Aged between 18–60 years old
- Based in Dhaka city
- Either part of the *hijra* community or belong to one of the stakeholder groups mentioned above
- Given their informed verbal consent to participate in the study

On the other hand, the exclusion criteria included those who did not consent to participate in the study, *hijra* who were not based in Dhaka city and those who were not in the physical or mental state to partake in the study.

### Study sites

The study will be conducted in Dhaka city, within four HIV prevention intervention service delivery points (known as Drop-in Centres) operated by icddr,b and funded by the Global Fund. The Drop-in Centres (DICs) are located in Badda, Tongi, Uttara and Darussalam. We are choosing these study sites because we have been working with this intervention for over two decades and have pre-established rapports with the service providers and, to some extent, members of the *hijra* community. Therefore, it would be easier to access the study community. As *hijra* reside within a socio-structural hierarchy consisting of a leader (*guru*) and her disciples (*chela*), they remain within tightly linked networks. Thus, we would be able to access the *chela* through the *guru*. The interviews will be conducted in the HIV prevention service centre premises, the HIV intervention outreach spots, the residences of *hijra* (*dera*) and other locations depending on the participants’ preference.

### Sample size and sampling techniques

The quantitative and qualitative components will apply separate sampling strategies. For the quantitative component, first-come-first serve sampling, a type of non-probabilistic sampling, will be adopted for a total sample size of 296 *hijra*. This sampling approach has been chosen given its convenience and feasibility for accommodating the mobile nature of the *hijra* community. As the *hijra* communities are occupied with various income-generating activities throughout the day such as blessing newborns, collecting money from marketplaces, etc., it would not be possible to prepare a sampling frame using simple random or other probabilistic sampling approaches within the stipulated data collection period. The sample size was calculated based on the available evidence about the SRHR-related rights indicators that will be applied in our study. For each of the indicators for *hijra*, sample size was calculated separately using the standard cross-sectional survey formula [27] with 5% precision, 95% confidence interval (CI) and design effect of 1.50. Finally, the maximum 296 was taken. The Table 3 shows the detailed breakdown of the sample size calculation based on the literature review of the outcome variables.

**Table 3:** Sample size calculation based on the literature review of the outcome variables.

Outcome value being measured	Value	Calculated sample size	Source article/country
Transgender women who have been denied healthcare services	19%	296	UNAIDS 2021
Transgender women who lack access to health information about their sexual health	93.8%	128	Reisner et al. 2010

On the other hand, the qualitative component will apply maximum variation sampling for the in-depth interviews (IDIs) and focus groups. This sampling approach is particularly beneficial for gaining a comprehensive understanding of the overall rights situation and identifying cross-cutting issues and discrepancies among diverse socio-demographic groups [28]. In this context, for selecting the IDI participants, we will develop a matrix enlisting the planned range of participants for each socio-demographic group, based on the available literature about different classifications of *hijra* used in previous research [28]. Based on this exercise, we plan to conduct 25–30 IDIs with *hijra*. As focus groups will be conducted with homogenous groups of *hijra* and HIV service providers, we plan to conduct 5–7 focus groups. To optimize the collection of authentic data, the groups will remain homogeneous, e.g. one focus group will exclusively consist of hijra or service providers. The groups will also be determined by the available literature on *hijra*. For the key-informant interviews (KIIs), we plan to formulate a list of participants which include *hijra* community leaders, healthcare practitioners, government and policy-level stakeholders, HIV intervention service providers, SRHR programme managers. For all qualitative interview types, the planned number of interviews is not static. Rather, it is dependent on points of data redundancy and saturation, whereby all forms of data collection will be stopped. Saturation will have been achieved when repeated data of the same content has been collected and analyzed.

### Phase 1: Evidence generation phase

#### Desk review

The evidence generation phase will start with the desk review. To gain a deeper understanding of the history, contexts and applications of the policy declaration, the research team will conduct a rigorous desk review. According to social science scholars, this step is crucial for understanding the nature of the pre-existing research, as well as research gaps that need to be addressed [29]. This is not an isolated exercise, rather this would serve as a complementary approach to be nestled throughout the data collection period. However, it would be beneficial to start with the desk review before deploying the researchers to the field, as this would help develop robust foundational knowledge which would subsequently guide them in their field activities. In the context of this study, the desk review will be integral not only for understanding the details about the third gender declaration but also for understanding how the policy is implemented in reality. To attain basic knowledge about the third gender declaration, the research team will read the relevant policy documents and any other historical discourse. To understand the existing grounded realities, the research team will peruse media reports about the adversities experienced by *hijra*, the existing local and global literature about the rights issues of *hijra*, and reports about relevant issues. This desk review will entail the reading of both published and unpublished literature, as this would help identify the actual knowledge gaps. The discrepancies between the policy declaration and the grounded reality will be identified by applying policy analysis (delineated in previous sections).

#### Quantitative data collection

The data collection will start by conducting quantitative surveys which will follow the semi-structured questionnaire format. The survey will pose questions on socio-demographic characteristics, sexual history/risk behaviours, SRHR knowledge, healthcare-seeking patterns for STIs and SRHR-related issues, sexual rights (i.e., autonomy of gender expression, choice of sexual partner and ability to negotiate safer sex behaviours), experiences of harassment, violation and other rights infringements. While most of the emphasis is on SRHR-related issues, as per the objectives, there are a few questions about their human rights-related agenda, namely their ability to exercise their rights in a mainstream forum beyond their *hijra* community. It is expected that quantitative data will not only allow us to respond to the research questions better by helping us understand the magnitude of SRHR burdens and SRHR-related rights infringements, but also guide us in identifying the priority areas to be emphasized during stakeholder discussions.

#### Qualitative data collection

The nuanced strengths of both the qualitative and quantitative approaches are expected to complement each other throughout the data collection process. While the quantitative component will garner a deeper understanding of the magnitude and priority areas, the qualitative approach will guide our understanding of the lived experiences of the *hijra*. Since the policy analysis framework entails the exploration of lived experiences, interpretative phenomenology will be used. According to qualitative scholars, interpretative phenomenology uncovers the meaning of a lived experience through in-depth reflective inquiry [30].

IDIs will be conducted to elicit in-depth information about lived experiences of the *hijra*, SRHR-related healthcare access barriers, challenges in exercising SRHR and other rights, and perspectives on how the Government can better respond to *hijra’*s needs. IDIs are particularly useful for gathering detailed information about people’s emotions, behaviours and perspectives; and often provide contextual information on other data (e.g. quantitative data), thus providing a comprehensive picture of the scenario [28]. Focus group discussions (FGDs), on the other hand, will explore societal issues, alongside shared and diverse perspectives about issues pertinent to the study objectives. The FGDs will not only be conducted with the *hijra* but also homogenous groups of service providers from the HIV prevention service centers. Each FGD will consist of 6–8 participants. Two researchers will be present during the discussion, one as a moderator who poses questions and mediates the discussion, and the other one as a notetaker of important issues and non-verbal communications.

Key-informant interviews will be conducted for collecting essential information from participants who are knowledgeable about *hijra*, or SRHR-related issues. The maximum intensity of sampling approach will be used which facilitates the selection of information-rich cases. The key-informants will include HIV prevention service providers (including peer educators), hijra guru and community-based organization leaders, healthcare providers at various layers of public healthcare facilities, experienced SRHR researchers and programme managers, policy makers and government stakeholders. It is expected that 1–2 key-informants will be selected from each group. The KIIs will deliberate about issues such as challenges in accessing healthcare and other basic citizenship rights, SRHR issues experienced by these populations, and recommendations for constructing a more gender-inclusive and sensitive policy for *hijra*. For particularly compelling cases, life case histories will be conducted. Qualitative scholars have acclaimed the use of life case histories for its ability to situate narrative accounts within the broader context of the personal, historical, social, political and institutional domains [31]. Life histories have a certain set of characteristics different from other qualitative methodologies, primarily its ability to explore multiple dimensions of a single person, whereas IDIs are more focused on a linear set of specific issues [31].

### Phase 2: Policy recommendation formulation

The findings from both the desk review and empirical mixed methods evidence will be analyzed using the policy analysis framework. Like the policy analysis framework, this will also be embedded as a conceptual framework throughout the study period, especially the second phase. The research team will synthesize these findings into a policy brief, which will contain the key findings in a palatable form that can be easily understood by stakeholders. The research team will facilitate series of meetings with a round-table of stakeholders and hijra community members which are listed in the study populations section. The hijra community members will also be included in order to ensure that the discussions and recommendations adequately capture their inputs and perspectives. During these meetings, policy recommendations, which also include a specific set of activities will be formulated in response to the quantitative rights-related indicators, alongside the lived experiences of *hijra* in relation to their SRHR and other rights. It is expected that these policy recommendations will be taken up by existing SRHR programs for *hijra* such as the HIV prevention interventions supported by the Global Fund and Government Health Sector. Moreover, these recommendations could be utilized by public and private healthcare modalities to facilitate a better mainstreaming response for *hijra*.

### Data analysis plan

Both quantitative and qualitative analysis will be conducted in relation to the policy analysis framework, where elements of the policy will be compared with the grounded realities presented by quantitative and qualitative data. For the quantitative component, statistical analysis will be performed using Stata version 13.0. Epi Info will be used for data entry. Range and consistency checks will be incorporated in the data entry screens. Thereafter, all data files will be converted to Excel for further cleaning by filtering to check the consistencies of denominators and responses to the survey questions. Descriptive statistics will be used to describe the indicators. All categorical variables will be expressed in terms of percentage points and numerical variables by mean with standard deviation for normally distributed variables, otherwise median will be used with inter quartile range.

As qualitative data collection and analysis are ongoing with reflexive processes [32], they will be conducted concurrently. This would allow for modifying interview guidelines, where necessary, and identifying data redundancies and saturation. Field team members will immediately transcribe recorded interviews. Although the interviews will be conducted in Bengali, it is assumed that the data will contain local dialects of the *hijra* community. Therefore, field researchers will carefully listen to the recorded data, explore and clarify the meanings of local phrases.

The research team will pursue the six steps of thematic analysis by Braun and Clarke [33]. The field team members will repeatedly peruse a small subset of the interview transcripts, and try to identify the themes and sub-themes. These themes will formulate the basis of a thematic matrix which will initially be applied to the remainder of the data. The contexts and meanings of these themes and sub-themes will be further analyzed. Qualitative data will be sorted by reading transcripts, and emerging and re-emerging issues which were not considered beforehand. These issues will be subsequently incorporated into interview guidelines and data gaps will be supplemented through ongoing field visits.

To ensure scientific rigor of the qualitative research, several approaches will be used, keeping in mind the principles of dependability, confirmability, transferability and credibility [34]. These four pillars constitute the qualitative analogue of validity and reliability in qualitative research. To achieve this, we will adopt triangulation, i.e., the use of multiple data collection sources, techniques, investigators and analytical approaches [28]. This approach is believed to corroborate the same phenomena that are widely occurring across various members of the hijra community, thus further increasing the credibility of the data [28]. In addition, peer debriefing sessions will be held to exchange and deliberate about findings and interpretations [35]. Moreover, member-checking sessions will be held to ensure the correct interpretation of the findings from the emic perspectives of the *hijra* [35].

### Ethics statement

We have attained the ethical approval from the Ethical Review Committee of icddr,b. Informed and understood verbal consent will be taken from participants before interviews. Their consent will be documented in the form of tick-boxes in the checklist provided on the consent form (this template is provided by the Ethical Review Committee of icddr,b). To maintain the confidentiality of the participant, there are no witnesses in the consent or interviewing process. There will be no written consent taken in order to protect the confidentiality of the *hijra* who may be reluctant to disclose their identities. The consent will be read out to the participants and after they have agreed to participate in the survey, the researcher will proceed with the interview.

While attaining the informed consent, the study objectives, methods, benefits and risks of the research will be explained to them. This study has the potential to benefit the broader hijra population by generating crucial insights which ultimately perpetuate policy-level recommendations. However, on the other hand, the proposed study carries its own set of risks. For instance, there is a chance that the participants could feel mentally distressed or traumatized as they have to share sensitive aspects of their lived experiences and challenges. Therefore, the participants were reassured at the time of reading the consent form of their right to decline responding to any questions, if needed, and to withdraw from the interview if they did not feel comfortable. However, the research team has prepared a contingency plan to navigate any instances of trauma or mental distress arising from the interviews. Specifically, since the team is part of a greater team that operates and manages HIV prevention interventions for hijra and other gender and sexually diverse people, they are networked with a body of community-based service providers both from the community and those with paraprofessional training who are trained to manage these cases of trauma and distress. Moreover, if the situation is severe, the HIV prevention interventions contain referral networks to professional psychologists and psychiatrists.

Specific data management techniques will be applied to ensure the protection of such confidential data. In particular, a unique identification number (ID) will be given to each study participant which will not be linked to their name. Utmost care will be taken to maintain confidentiality of the information collected. All questionnaires will be kept in locked cabinets in the office which will be accessible to the investigators and field researchers. All computers containing data will be password protected. All interviews will be conducted in a private space where the participant is comfortable. All of the members of the research team, specifically those who are conducting the interviews and handling the transcriptions, will be trained on the steps for maintaining confidentiality during data collection and management. All of the research team members have been sensitized beforehand about the complexities and cultural sensitivities of hijra, and many of these team members have also previously worked with hijra and other gender and sexually diverse people in both research and programmatic capacities. Therefore, this considerably minimizes the risk of the participants being exposed to stigma and discrimination.

## Timeline of the study

As mentioned in the previous section, the study protocol has received the ethical approval from the Ethical Review Committee of icddr,b. The staff have been recruited for the study, and trained on the issues relevant to the study. The data collection is yet to be started. The timeline is specified in Table 4.

**Table 4:** Timeline of the proposed study.

Planned activity	Apr 2023	May 2023	Jun 2023	Jul 2023	Aug 2023	Sep 2023	Oct 2023	Nov 2023	Dec 2023	Jan 2024	Feb 2024	Mar 2024
Recruitment and training												
Field testing and finalization of data collection tools												
**Phase 1: Evidence generation**												
Comprehensive desk review												
Data collection (quantitative)												
Data entry, cleaning and analysis												
Data collection (qualitative)												
Data analysis (qualitative)												
**Phase 2: Policy recommendation formulation**												
Packaging of lay summaries												
Policy stakeholder meetings												
Report writing (phase 1)												
Report writing (phase 2)												
Manuscript writing												
Dissemination												

## Discussion

This study applies two scientifically acclaimed conceptual frameworks which will help guide the analysis of the gender policy, as well as engage stakeholders in a participatory manner. By introducing a stakeholder engagement component, we are ensuring an active policy translation component. Moreover, this study covers an uncharted territory of rights in a domain where transgender women are typically known in Bangladesh and beyond for their HIV transmission potential. However, this study consists of various activities which may necessitate substantial efforts and time to complete. Moreover, another limitation of this study is that it merely goes up to the stage of devising policy recommendations, rather than formulating an intervention design.

Several dissemination initiatives will be taken throughout and after the study. In addition to distributing policy briefs with the stakeholders during the policy recommendation formulation phase, we plan to conduct a dissemination seminar with the stakeholders and members of the scientific community once both phases are complete. Additionally, we plan to publish a study report to be shared with stakeholders, program implementers and the research community. This will be coupled with a few scientific articles to be submitted to international peer-reviewed journals.

### Statement about prisoners

There were no participants living in prisons in this study, therefore this statement is not applicable.

### Authors’ information

Programme for HIV and AIDS, Health Systems and Population Studies Division (HSPSD), icddr,b.
